# Hampton’s Hump—A Rare Radiological Feature in Patients with Pulmonary Embolism in a Single-Center Study

**DOI:** 10.3390/jcm14061900

**Published:** 2025-03-12

**Authors:** Kinga Kubiak, Katarzyna Bazylewicz-Zakrzewska, Wojciech Poncyljusz

**Affiliations:** Department of Diagnostic Imaging and Interventional Radiology, Pomeranian Medical University in Szczecin, 71-252 Szczecin, Poland

**Keywords:** Hampton’s hump, pulmonary embolism, CTA

## Abstract

**Background**: Pulmonary embolism is a potentially life-threatening condition caused by the sudden occlusion of pulmonary arteries. Its non-specific and highly variable clinical presentation often leads to undiagnosed cases, making computed tomography pulmonary angiography (CTPA) essential for diagnosis. Hampton’s hump is a rare radiological finding associated with pulmonary embolism, characterized by a wedge-shaped, pleural-based opacification due to pulmonary infarction. **Methods**: This study examines the correlation between pulmonary embolism severity and the presence of Hampton’s hump in CTPA based on a database of 428 patients. **Results**: We observed a correlation between the presence of Hampton’s hump and an increased likelihood of rider-type embolism, pleural effusion, and dilation of the pulmonary trunk and left pulmonary artery. The data indicate that patients with at least one risk factor for severe pulmonary embolism are more likely to present with Hampton’s hump. Furthermore, the presence of this sign in patients diagnosed with pulmonary embolism and pleural effusion substantially increases the risk of in-hospital death. **Conclusions**: This study underscores the significance of Hampton’s hump as a rare but clinically relevant radiological finding in patients with pulmonary embolism. Given the limited number of studies on this topic, our findings provide valuable insights into the role of Hampton’s hump in the context of pulmonary embolism.

## 1. Introduction

Pulmonary embolism (PE) is a prevalent and potentially life-threatening condition with multiple etiologies and diverse clinical manifestations. It results from the obstruction or constriction of the pulmonary artery or its branches by embolic material. PE ranks as the third most common acute cardiovascular condition, following myocardial infarction and stroke. In the general population, mortality depends on PE severity, with the risk of death within 90 days reaching up to 50% in patients with massive PE (accompanied by systemic arterial hypotension) [1]. The annual prevalence of this life-threatening condition is approximately 60–70 cases per 100,000 individuals [2]. PE is predominantly caused by a thrombus that migrates from the deep veins of the pelvis and lower extremities into the pulmonary arterial circulation via the venous system [3]. Up to 50% of venous thrombi in the lower extremities eventually embolize, with a higher prevalence among those located above the knee [4,5]. Hemodynamic instability may result from obstruction at the bifurcation of the main pulmonary arteries by large clots, while lung infarction and pleuritic pain may occur due to the distal migration of smaller clots. Non-thrombotic pulmonary embolism (NTPE) includes septic, hydatid cyst, fat, tumor, gas, polymethyl methacrylate (bone cement), amniotic fluid, and foreign body embolism [6].

Breathlessness, pleuritic chest pain, anxiety, and coughing are frequently reported symptoms of pulmonary embolism (PE) [7]. However, these symptoms are not specific to PE and can occur in other conditions, including acute coronary syndrome, thoracic aortic dissection, pneumothorax, and pneumonia. Many patients presenting to the emergency department with these symptoms undergo evaluation for PE. In patients with a positive D-dimer test or a high pretest probability, chest imaging with CTPA should be performed [8].

Diagnosing PE is challenging due to the absence of a pathognomonic sign or symptom. In patients with PE involving the main or lobar pulmonary arteries, the classic symptoms of dyspnea or tachypnea are present in 92% of cases [9]. However, in patients with smaller subsegmental pulmonary embolisms, only 73% experience dyspnea, 70% present with tachypnea, and 66% report pleuritic chest pain [10]. According to recent studies, fewer than 20% of individuals evaluated for suspected PE are ultimately diagnosed with the disease [11]. Certain groups are at higher risk for PE and venous thromboembolism (VTE), including oncology patients, individuals with thrombophilia, immobilized patients post-surgery or -trauma, pregnant and postpartum women, women using oral contraceptives (OCs) or hormone replacement therapy (HRT), and individuals with a history of long-haul air travel [12]. Historically, advanced age has been considered a risk factor for PE. However, recent findings suggest that younger patients may be at greater risk due to less-developed collateral circulation and elevated endogenous nitric oxide levels, which contribute to increased vascular anastomoses and bronchial blood flow [13,14].

In patients with suspected PE, computed tomography pulmonary angiography (CTPA) is considered the first-line diagnostic procedure, with sensitivity and specificity ranging from 89 to 98% and 96 to 100%, respectively [15]. Although lung perfusion scintigraphy is a useful tool for detecting PE, it is rarely employed as a first-line diagnostic approach in acute settings due to limited availability in emergency departments and the time required for examination. Ultrasound with a compression test is a simple and rapid method for detecting venous thrombosis in the lower extremities. The widespread availability of ultrasound machines in hospitals, combined with the relatively short examination time, has made it a key diagnostic tool for detecting venous thromboembolism (VTE) and indirectly predicting the likelihood of PE. However, as up to 50% of PE patients do not exhibit VTE on imaging, a negative venous ultrasound alone is insufficient to rule out PE [3]. Additional diagnostic techniques for PE include laboratory testing (specifically D-dimer levels), electrocardiography (ECG), echocardiography (UCG), and pulmonary arteriography.

One radiological sign associated with pulmonary embolism is Hampton’s hump, named after Aubrey Hampton and Benjamin Castleman, who first described it in 1940 [16,17]. Scientists analyzed autopsy series to determine the locations of opacities observed on chest radiographs of patients with PE and compared them to cases of pulmonary infarction confirmed at autopsy [18,19].

**Figure 1:**
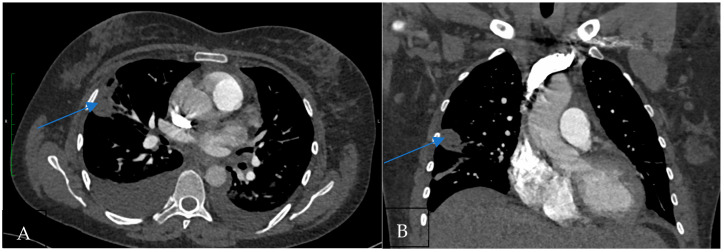
(**A**) CT pulmonary angiogram shows the wedge-shaped subpleural pulmonary infarction in the axial plane (Hampton’s hump; arrow). (**B**) CT pulmonary angiogram shows the wedge-shaped subpleural pulmonary infarction in the coronal plane.

Hampton’s hump (Figure 1) is a radiological finding characterized by a wedge-shaped, peripheral opacification, signifying pulmonary infarction distal to a pulmonary embolus. It is important to emphasize that Hampton’s hump specifically indicates pulmonary infarction secondary to PE rather than PE itself [16].

Only a few publications examine the “Hampton hump” in depth, making it an underrepresented topic in the academic literature. The lack of extensive research on this topic presents a significant gap in the field, limiting the knowledge of its significance. The purpose of this article is to address the Hampton hump’s underexplored status and provide a full study of its relevance and possible impact. By exploring this understudied topic, we hope to not only address a knowledge gap but also emphasize the need for additional research to properly understand the larger ramifications. Our analysis of this issue is meant to generate scholarly debate and promote subsequent research on the subject.

## 2. Materials and Methods

This study was a single-center retrospective analysis of collected data. Inclusion criteria included symptomatic and asymptomatic patients who underwent CT in pulmonary embolism protocol in our department between 1 January 2020 and 31 December 2022. The age and sex of each patient were documented. Two radiologists independently reviewed all the images, evaluating the imaging findings of all participants, including infarct morphology and metabolic pattern, involvement of pulmonary arteries, and symptoms associated with affected pulmonary lobes and pleural effusion. The criteria used to diagnose acute PE were limited to identification of the trailing edge of a thrombus obstructing an artery or identification of an intraluminal filling defect; exclusion of acute FE was based on the absence of these findings. Consultation was used to resolve the interpretational differences. If readers were unable to clarify discrepancies between imaging results, the case was excluded. Analyses were performed on a minimum of two reconstruction planes, often axial and coronal. CT scans were ordered by physicians caring for the patients.

Chest CT scans were performed using one of three commercial 64-row 128-slice CT scanners (Siemens Healthcare Somatom Definition Edge, Forchheim, Germany) at our site. CT acquisition parameters were 128 × 0.6 mm sections with a thickness of 0.75 mm and pitch factor of 1.2, ranging from 80 to 130 to 70 to 140 kilovolts and ≥345–800 mA. Rotation time was 0.5 s. All examinations were performed with 80 mL of non-ionic contrast agent Iomeron 400 mg/mL at an average rate of 4 mL per second as per our protocol. Scanning was triggered when the average Hounsfield unit of the pulmonary artery reached 80 HU. Image reconstruction included contiguous 0.75 mm thick sections with high resolution and standard algorithms for evaluation of the lung parenchyma and mediastinum. Patients were examined using the single-breath-hold technique.

The aim was to determine the prevalence of Hampton’s hump in patients with pulmonary embolism detected on computed tomography pulmonary angiography (CTPA) and to examine the correlation between the presence of Hampton’s hump and the severity of pulmonary embolism.

## 3. Results

The study cohort comprised 428 patients diagnosed with pulmonary embolism between 1 January 2020 and 31 December 2022. The average age was 66 years (range 14–97 years), and 54% of patients were men. The study group included both symptomatic and asymptomatic patients. Table 1 summarizes the patients’ characteristics.

Among the study population, 2% of patients had massive thromboembolic disease with saddle pulmonary embolism, pleural effusion was found in 31% of patients, dilation of the pulmonary trunk was observed in 35% of patients, and dilation of the proximal part of the right and left pulmonary arteries was present in 43% and 18% of patients, respectively. Patients with Hampton’s hump accounted for 8% of all pulmonary embolism cases.

In the Hampton’s hump subgroup, the average age was 64 years (range 28–93 years), the majority of patients (51%) were men, and dilation of the pulmonary trunk and the proximal parts of the right and left pulmonary arteries was observed in 40%, 43%, and 20% of patients, respectively. Pleural effusion was detected in 63% of individuals, and saddle pulmonary embolism was present in 6% of patients with Hampton’s hump.

Our analysis indicates no significant association between patient age and the occurrence of Hampton’s hump in pulmonary embolism. The average age of patients with pulmonary embolism was 66 years, whereas the average age of those with Hampton’s hump on CTPA was 64 years. In both groups, the predominant gender was male, accounting for 54% and 51%, respectively.

However, patients with pleural effusion or saddle pulmonary embolism were more likely to have Hampton’s hump (31% vs. 63% and 2% vs. 6%, respectively). A diagnosis of oncological disease was confirmed in 31% of all patients. Among patients exhibiting Hampton’s hump, 26% had an oncological illness.

Our study found that the in-hospital mortality rate was 7% (27 deaths) among patients with pulmonary embolism (PE) and pleural effusion, compared to 5% (21 deaths) in patients with PE but without pleural effusion or Hampton’s hump. In-hospital mortality for patients with PE, pleural effusion, and Hampton’s hump was 11% (four deaths).

Saddle pulmonary embolism is an uncommon occurrence. In our study, saddle embolism was found in 6% of patients with Hampton’s hump and in 8% of those without the sign. Among patients with PE but without Hampton’s hump, the in-hospital mortality rate for saddle pulmonary embolism was 25%. Notably, none of the patients with Hampton’s hump who died had saddle embolism.

Additionally, oncology patients have a higher risk of mortality from pulmonary embolism. Our study indicates that the in-hospital mortality rate for cancer patients with PE was 5% (21 deaths). Among patients with PE and oncological disease, 5% (20 deaths) occurred in those without Hampton’s hump, while 3% (1 death) occurred in patients with Hampton’s hump.

Our study found an in-hospital mortality rate of 12% for patients without Hampton’s hump and 11% for those with it. Additionally, several patients who died from embolism had severe comorbidities. One patient presented with multi-organ injury, another had attempted suicide, and a third had a diagnosed case of neurosarcoidosis. The remaining patients had suffered a stroke.

## 4. Discussion

The diagnosis of pulmonary embolism remains challenging. Before the advent of advanced imaging techniques, chest radiographs were the primary modality used for diagnosing pulmonary embolism. Currently, however, chest radiographs are believed to provide insufficient detail to confirm or exclude the diagnosis of acute pulmonary embolism (PE).

Nowadays, CTPA is the gold standard for detecting PE which offers several advantages, including widespread availability, minimal invasiveness, and rapid scan duration. Furthermore, CTPA can also diagnose other conditions, such as pneumonia, rib fractures, or pneumothorax. Therefore, it is essential to understand the various imaging findings that support the diagnosis of PE on CTPA [20].

Hampton’s hump is an uncommon, pleural-based, wedge-shaped opacity that indicates infarcted lung parenchyma [21]. Pulmonary infarction develops when pulmonary perfusion is decreased. Normal lung tissue receives a dual blood supply from both the pulmonary arterial circulation and the bronchial arterial circulation. Pulmonary infarction is rare due to the physiological anastomosis between the bronchial arteries and the ischemic zone, which increases blood flow to the affected area when pulmonary artery function is impaired [22,23]. However, if both arterial supply pathways are occluded, the absence of reperfusion leads to pulmonary infarction. Tsao et al. found in autopsy studies that emboli smaller than 3 mm in the distal pulmonary artery significantly increase the risk of pulmonary infarction [23]. Kirchner et al. demonstrated that the incidence of pulmonary infarction is negatively correlated with the distance from an embolus in the pulmonary artery to the pleura [24]. These findings suggest that obstruction of distal vessels is the primary cause of pulmonary infarction.

In patients diagnosed with PE, follow-up autopsies have revealed pulmonary infarction in 15–31% of cases, while computed tomography has identified pulmonary infarction in 9–36% of patients. Hampton’s hump has been found to have moderate specificity for diagnosing PE, although its sensitivity is limited [23,25,26]. A study analyzing radiographs from the multicenter Prospective Investigation of Pulmonary Embolism Diagnosis (PIOPED) trial reported that Hampton’s hump had a sensitivity of 22% and a specificity of 82% [21].

We discovered that age could be a predictor of in-hospital mortality. In our research, the mean age of patients who died was 70.25 years (range 48–93). Among patients with Hampton’s hump, it was 80.75 years, with a range of 63 to 93. Older individuals encountered an increased risk of complications and less favorable results subsequent to pulmonary embolism.

In our group, the mortality rates for male and female patients were similar, suggesting that there was no significant association between sex and in-hospital mortality.

Recent cohort studies have demonstrated a correlation between the presence of pleural effusion and higher mortality rates in patients with pulmonary embolism (PE). Specifically, the in-hospital mortality rate for PE with pleural effusion was 12.0%, compared to 4.3% for PE without pleural effusion [27]. Our study found that 31% of PE patients had pleural effusion, and 51% of patients who died had this condition. In comparison, a previous study reported that 41.2% of patients with PE had pleural effusion [28].

Additionally, in the cited study, the in-hospital mortality rate for saddle pulmonary embolism was 9.2% [27]. In our group, 20% of patients with saddle pulmonary embolism died.

Furthermore, we observed that pulmonary trunk dilation and left pulmonary artery dilation were more frequently found in patients with Hampton’s hump. These findings suggest that Hampton’s hump may be associated with more severe cases of pulmonary embolism. However, the dilatation of the pulmonary trunk, proximal right pulmonary artery, and proximal left pulmonary artery were not statistically significant predictors of in-hospital mortality in this investigation.

The overall in-hospital death rate for the entire group was 12% (52 of 428 patients). The death rate among patients with Hampton’s hump was 11% (4 out of 35 patients), although this difference was not statistically significant. This indicates that although Hampton’s hump may correlate with more severe pulmonary embolism, its presence does not significantly impact the short-term survival rate within the total group. However, in patients with multiple indicators of severe PE, such as pleural effusion and Hampton’s hump, the in-hospital mortality rate was 100%. Our analysis suggests that patients with at least one risk factor for severe PE are more likely to exhibit Hampton’s hump. Additionally, the presence of this sign in PE patients with pleural effusion significantly increases the risk of in-hospital death. Interestingly, among oncology patients, Hampton’s hump was observed less frequently.

The purpose of this article is to highlight the rarity of Hampton’s hump and encourage further research on the topic. However, the study of Hampton’s hump encounters multiple limitations that hinder a comprehensive understanding of the condition. Firstly, there is a lack of comprehensive literature systematically investigating Hampton’s hump across diverse patient populations, limiting the generalizability of current findings. Most existing studies rely on individual case reports or small case series, making it difficult to draw definitive conclusions regarding its prevalence, diagnostic accuracy, and clinical significance. Another limitation is the inconsistent use of imaging modalities. Various studies utilize different radiological techniques, such as chest X-rays, CT scans, and MRI, which complicates comparisons between cases. Additionally, differences in sensitivity and specificity across these imaging methods may result in missed diagnoses.

The study found numerous significant indicators of in-hospital death in individuals with pulmonary embolism. Hampton’s hump, pleural effusion, and advanced age were all correlated with an elevated mortality risk. These findings highlight the significance of identifying Hampton’s hump as a possible indicator of more severe PE. Nonetheless, although linked to increased mortality, Hampton’s hump did not prove to be the primary predictor; other variables, including saddle pulmonary embolism and pleural effusion, were associated with poor outcomes. The cohort of Hampton’s hump patients was limited, and more research is needed to better understand the significance of Hampton’s hump in the prognosis of PE and the clinical impact of Hampton’s hump.

## Figures and Tables

**Table 1:** The patients’ characteristics.

	All of the Patients	Patients with Hampton’s Hump
Average age (range)	66 (14–97)	64 (28–93)
Sex (male/female)	230:198 (54%:46%)	18:17 (51%:49%)
Saddle pulmonary embolism	8 (2%)	2 (6%)
Pleural effusion	135 (31%)	22 (63%)
Hampton’s hump sign	35 (8%)	
Dilatation of the pulmonary trunk	149 (35%)	14 (40%)
Dilatation proximal part of the right pulmonary artery	184 (43%)	15 (43%)
Dilatation proximal part of the left pulmonary artery	77 (18%)	7 (20%)
Oncological disease	131 (31%)	9 (26%)
In-hospital mortality rate	52 (12%)	4 (11%)

## Data Availability

The original contributions presented in this study are included in the article. Further inquiries can be directed to the corresponding author.

## Abbreviations

The following abbreviations are used in this manuscript:

CTPA	computed tomography pulmonary angiography
PE	pulmonary embolism
VTE	venous thromboembolism
ECG	electrocardiography
UCG	echocardiography
NTPE	non-thrombotic pulmonary embolism
